# Examining chronic care patient preferences for involvement in health‐care decision making: the case of Parkinson's disease patients in a patient‐centred clinic

**DOI:** 10.1111/hex.12497

**Published:** 2016-09-14

**Authors:** Natalie Zizzo, Emily Bell, Anne‐Louise Lafontaine, Eric Racine

**Affiliations:** ^1^ Neuroethics Research Unit Institut de recherches cliniques de Montréal Montreal QC Canada; ^2^ Division of Experimental Medicine and Biomedical Ethics Unit McGill University Montreal QC Canada; ^3^ Department of Neurology and Neurosurgery McGill University Montreal QC Canada; ^4^ McGill University Health Centre Montreal QC Canada; ^5^ Department of Medicine Université de Montréal Montreal QC Canada; ^6^ Department of Social and Preventive Medicine Université de Montréal Montreal QC Canada

**Keywords:** bioethics, decision making, Parkinson's disease, patient preferences, patient‐centred care, patient–physician relationship, relational autonomy

## Abstract

**Background:**

Patient‐centred care is a recommended model of care for Parkinson's disease (PD). It aims to provide care that is respectful and responsive to patient preferences, values and perspectives. Provision of patient‐centred care should entail considering how patients want to be involved in their care.

**Objective:**

To understand the participation preferences of patients with PD from a patient‐centred care clinic in health‐care decision‐making processes.

**Design, setting and participants:**

Mixed‐methods study with early‐stage Parkinson's disease patients from a patient‐centred care clinic. Study involved a modified Autonomy Preference Index survey (N=65) and qualitative, semi‐structured in‐depth interviews, analysed using thematic qualitative content analysis (N=20, purposefully selected from survey participants). Interviews examined (i) the patient preferences for involvement in health‐care decision making; (ii) patient perspectives on the patient–physician relationship; and (iii) patient preferences for communication of information relevant to decision making.

**Results:**

Preferences for participation in decision making varied between individuals and also within individuals depending on decision type, relational and contextual factors. Patients had high preferences for communication of information, but with acknowledged limits. The importance of communication in the patient–physician relationship was emphasized.

**Discussion:**

Patient preferences for involvement in decision making are dynamic and support shared decision making. Relational autonomy corresponds to how patients envision their participation in decision making. Clinicians may need to assess patient preferences on an on‐going basis.

**Conclusion:**

Our results highlight the complexities of decision‐making processes. Improved understanding of individual preferences could enhance respect for persons and make for patient‐centred care that is truly respectful of individual patients’ wants, needs and values.

## 1 Introduction

Parkinson's disease (PD) is a chronic neurodegenerative disease, affecting an estimated 7–10 million individuals worldwide.^1^ It causes progressive impairments in motor control and often includes psychiatric and cognitive comorbidities in patients as the disease advances. There has been a call for the treatment of PD to be delivered within a patient‐centred model of care.^2^ Although there are many definitions of patient‐centred care (PCC), it has been broadly conceived of as “respectful of and responsive to individual patients’ preferences, needs, and values,”^3^ and it aims to have patient values guide clinical decision making.^3^ PCC is endorsed by both the Institute of Medicine^3^ and the Canadian Medical Association^4^ and has been shown to have instrumental value, with tangible benefits that include better health outcomes.^5^, ^6^ Furthermore, PCC has been supported from an ethical standpoint;^7^ it is viewed as an extension of the principles of respect for persons and for autonomy.

However, implementing PCC can prove to be challenging, as there is a current lack of understanding on the perspectives, values and preferences of patients with PD for care.^8^, ^9^, ^10^, ^11^ In particular, how patients want to be involved in decision‐making processes remains unclear.^12^, ^13^, ^14^ In fact, investigations into the variability of patient preferences for the decision‐making process show that physicians misjudge patient desire for involvement in decision making.^13^ This research points to a need to improve the understanding of PD patient preferences for involvement in care. While there has been a great deal of quantitative investigations into these preferences, the qualitative literature is far more sparse^15^ and patient preferences for involvement in care have not been investigated, to our knowledge, in a chronic care neurodegenerative population.

Consequently, we conducted both quantitative surveys and qualitative interviews with PD patients in a PCC clinic to investigate their preferences for involvement in health‐care decision‐making processes. In our analysis, we draw on the analytic stages of decision making proposed by Charles et al.,^16^ including (i) information exchange, (ii) deliberation and (iii) decisional control, and consider how patient preferences for involvement may vary with each of these stages. Our results reveal the complexity of decision‐making preferences in a PD population and can provide insight for the provision of PCC to other chronic illness and neurodegenerative populations.

## 2 Methods

The sample population in this study consisted of early‐stage PD patients from a PCC movement disorder clinic. Patients who were not proficient in English or French, or who had cognitive deficits based on validated cognitive tests (Montreal Cognitive Assessment) on file, were excluded, as these factors could impact their ability to be involved in decision making. All appropriate candidates, as identified by medical staff at the clinic, were approached to complete a modified version of the Autonomy Preference Index (API).^17^ Survey participants self‐identified whether they were interested in participating in a follow‐up qualitative semi‐structured interview. Interview participants were selected by maximum variation sampling, a form of purposeful sampling, to maximize the diversity of participants based on age, gender and educational background.^18^ Interviews were conducted approximately 4–16 weeks after the completion of surveys. The authors’ institutional research ethics boards approved the research protocol, and all participants gave their free and informed written consent.

### 2.1 Quantitative methods

Participants were surveyed in their preferred language (English or French) using a modified version of the API. The API consists of (i) a six‐item decision‐making scale, which measures general desire to participate in medical decisions; (ii) an eight‐item information scale, which measures desire for medical information; and (iii) five vignettes, which measures desire to participate in medical decisions in specific clinical scenarios. For the decision‐making and information scales, summed scores were transformed to a range of 0–100, where a higher score indicates higher preferences for decision‐making involvement and for receipt of information, respectively. The vignettes featured in the API are designed to elicit decision‐making preferences in the context of different levels of illness severity. The mild (upper respiratory tract illness) and moderate (hypertension) disease vignettes came from the standard API and were kept because of their applicability and relevance. Three novel vignettes were generated to represent the severe disease in the specific context of PD to ensure greater relevance to our participants. The PD‐specific vignettes were developed in collaboration with an interdisciplinary PD medical team to feature PD progression (mild worsening of symptoms, moderate worsening of symptoms, new appearance of psychological symptoms). In each vignette, participants indicated their preferred decision‐maker for three decisions (decision‐making scores: 1 = doctor alone; 2 = mostly the doctor; 3 = the doctor and you; 4 = mostly you; 5 = you alone). For each vignette, the decision‐making scores were summed and transformed to a range of 0–10, where a higher score indicated greater desire for involvement in the decision‐making process.

Descriptive statistics (means, frequencies, range and standard deviation) were calculated for the sample on each scale of the API and for each vignette. The influence of demographic factors (age, gender, level of education) and medical information preferences (information scale of the API) on decision‐making preferences as a final outcome measure (decision‐making scale of the API) was examined using univariate and multivariable logistic regression analyses. Bivariate relationships between the transformed decision‐making preference scores on the five vignettes were examined using Pearson's correlation analysis. Repeated‐measures ANOVA mixed‐model approach assessed whether the vignettes had an effect on the final transformed decision‐making score on this part of the survey. Pre‐planned post hoc analysis compared the average decision‐making scores on the general medical vignettes to each of the three PD vignettes. The average decision‐making scores of the three PD‐specific medical scenarios were also compared. All statistical analyses were carried out using SAS 9.4 statistical software. For all inferential analyses, the probability of type 1 error was a priori fixed at alpha=0.05.

### 2.2 Qualitative methods

We aimed to interview approximately 20 patients, as we expected this N was sufficient for theoretical saturation (i.e. the point at which there was no significant new data). Indeed, this N was consistent with similar qualitative research,^11^, ^19^, ^20^ and an N of 20 yielded sufficiently dense data with diverse experiences and perspectives represented. If there was a partner or another individual who regularly attended clinical appointments with the patient, the patient was invited to include this person in the interview as this allowed us to acquire ecologically valuable data (i.e. a more natural representation of the decision‐making process).^21^

Interviews were semi‐structured with open‐ended questions centred on our research aim. We examined (i) patient preferences for involvement in health‐care decision making; (ii) patient perspectives on the patient–physician relationship, which is central to health‐care decision making; and (iii) patient preferences for the communication of information. Our questions on participation preferences did not specify the types of health‐care decisions, which allowed patients to interpret the question to the types of decisions most representative of their experience. We also inquired about two hypothetical situations: one in which there is a conflict between patient and physician and one in which the patient might be excluded from decision making. When patients’ partners were present, questions were modified to include their perspectives.

Interviews were conducted face‐to‐face, and were held at the primary author's research institution or at the specialist clinic, according to the patient's preference. The interviews were conducted in English or French, audio‐recorded, transcribed verbatim by an external professional transcription service and verified by a team member. A technical error resulted in failure to record one interview; in this case, detailed notes were taken immediately after the interview and verified by the participant for their accuracy, and then, these notes were used for analysis.

We analysed (“coded”) interview transcripts using thematic qualitative content analysis.^22^ An initial coding guide was developed after review of transcripts and a team discussion. This coding guide was piloted on a diversified sample of interviews (N=5, or 25% of total sample). Pilot results led us to revise the coding guide, update coding of the initial sample and code the remainder of interviews. The coding guide contained definitions and rules for the application of each code to ensure rigour and thoroughness. Upon the completion of coding, the results were reviewed and some nodes were further analysed. The primary author conducted the interviews and coded all interviews; the second author systematically reviewed all coding. Disagreements between the coder and the reviewer were discussed to achieve consensus, and the last author arbitrated outstanding disagreements. Coding was supported by the QSR NVivo 9 qualitative analysis software package. The final key themes for coding were as follows: (i) preferred decision‐making model (e.g. how should each individual be involved in decision making?); (ii) qualities of a good patient–physician relationship (e.g. general features of a good relationship, important qualities of a patient and of a physician, values important to care); (iii) PD information preferences (e.g. sources of and desire for information, limits to learning information).

Qualitative content is summarized and direct quotes are used to illustrate the perspectives of participants. Some quotes reported in this study were translated from French to English by the primary author and verified by another bilingual team member (the last author). Participant's names and identifying details have been removed to protect confidentiality. Patients are identified in text by the letter P followed by a number that was assigned sequentially as surveys were completed. Patients’ partners share the same identifier as the patient but are differentiated by a prime symbol (e.g. PXX′), and the letter “I” identifies the interviewer. Some quotes contain minor edits to enhance readability.

## 3 Results

### 3.1 Quantitative results

#### 3.1.1 Participant demographics

Sixty‐five patients with PD completed the survey, 27 females (41.5%) and 38 males (58.5%). The age range of participants was 39–85, with a mean age of 68. Twenty‐one participants (33.9%) had an education that was equal to or less than a professional college degree, 20 participants (30.8%) had a bachelor's degree and 24 patients had postgraduate education (36.9%).

#### 3.1.2 Decision‐making preferences

The mean decision‐making scale score was 62.8±16.6 out of a possible range of 0–100 (range: 20–97). Distributions of responses to statements on the decision‐making scale are presented in Table 1. For all questions except Q5 and Q6, the highest percentage of participants chose the answer indicating the most autonomous preference. Responses for questions five and six indicated that most participants desired less autonomy in these scenarios; only two participants preferred high autonomy answers for both of these questions.

**Table 1:** Distribution of responses to statements describing decision‐making preferences (% of participants responding)^a^

	Strongly disagree				Strongly agree
Q1: The important medical decisions should be made by your doctor not you	33.9	6.2	23.1	15.4	21.5
Q2: You should go along with your doctor's advice even if you disagree with it	29.2	16.9	20.0	16.9	16.9
Q3: When hospitalized you should not be making decisions about your own care	32.3	26.2	13.9	7.7	20.0
Q4: You should feel free to make decisions about everyday medical problems	6.2	7.7	23.1	18.5	44.6
**Q5: If you were sick, as your illness became worse you would want your doctor to take greater control**	9.2	12.3	21.5	26.2	30.8
Q6: You should decide how frequently you need a check‐up	27.7	16.9	30.8	7.7	16.9

a Shaded cells indicate what would be the response aligning with a preference for greatest autonomy in medical decision making.

#### 3.1.3 Information preferences

The mean information seeking scale score was 87.9±14.9 out of a possible range of 0–100 (range: 28–100). The mode was 100, with 17 participants (26%) scoring the maximum on information preferences.

#### 3.1.4 Vignette decision‐making preferences

Mean decision‐making scores for each vignette are presented on a possible range of 1–10 in Table 2. Decision‐making scores in vignettes 2 and 3, and vignettes 3 and 4, were moderately correlated (vignettes 2 and 3: *r*=.50, 95% CI [0.29; 0.66] *P*=<.0001; vignettes 3 and 4: *r*=.50, 95% CI [0.30; 0.67] *P*=<.0001). There was a significant effect of vignette score on decision‐making scores, as measured through a repeated‐measures ANOVA (*P*<.001). When their means were compared, there was a significant difference between decision‐making scores in the general medical vignettes (vignettes 1 and 2) and in each of the PD‐specific vignettes (vignette 3 (estimate=1.10, 95% CI [0.74; 1.46], *P*=<.0001), vignette 4 (estimate=0.55 (95% CI [0.155; 0.937]), *P*=.007) and vignette 5 (estimate=−1.18 (95% CI [−1.618; −0.736]), *P*=<.0001). Significant differences were also observed in comparing the means of decision‐making scores between vignettes 3, 4 and 5, with decision‐making scores increasing with disease severity (vignettes 3 and 4 (estimate=−0.55 (95% CI [−0.910; −0.198]), *P*=.003); vignettes 4 and 5 (estimate=−1.72 (95% CI [−2.216; −1.23], *P*=<.001); vignettes 3 and 5 (estimate=−2.28 (95% CI [−2.773; −1.780], *P*=<.0001).

**Table 2:** Mean API score in different vignettes

	Vignette 1 (cold)	Vignette 2 (blood pressure)	Vignette 3 (routine PD)	Vignette 4 (PD progression)	Vignette 5 (PD‐associated emotional distress)
Mean (potential range of 0–10)	5.9 (SD ±1.59)	5.0 (SD ±1.52)	4.4 (SD ±1.39)	4.9 (SD ±1.49)	6.7 (SD ±1.46)
Range	3–9	2–9	2–7	2–7	3–10

#### 3.1.5 Univariate and multivariable logistic regression analyses

We examined the influence of four explanatory variables on preference for decision‐making scores (decision‐making scale of the API) through univariate and multivariable logistic regression analyses. The four explanatory variables were identified a priori and included age (continuous variable), gender (M, F), education (≤professional college, bachelors university, graduate) and API information scale score (continuous variable). Listwise deletion was applied for all explanatory variables, resulting in the exclusion of one subject from regression analyses due to missing age in the database. Thus, the linear regression models were carried out on 64 participants. Overall, none of the factors reached statistically significant levels in the multivariate or univariable models.

### 3.2 Qualitative results

#### 3.2.1 Participant demographics

Twenty PD patient participants were interviewed, 10 males and 10 females. Participants were aged 50–77, with a mean age of 63. Fifteen interviews were conducted in English, and five were conducted in French. The average length of time participants were patients in the specialist clinic was 3 years, with 6 months as the shortest time and 7 years as the longest. Seven patient participants had a professional college education or less, four had university bachelor's degrees, and nine held graduate degrees. In four instances, patients’ partners were consented to and present for the interview; in two of these interviews, the partners contributed significantly.

#### 3.2.2 Patient preferences for involvement in health‐care decision making

##### Patients prefer involvement in decision making

In discussing decision making, patients emphasized the need for communication of information and preferences between physician and patient, with the deliberation of treatment options. In general, patients found it acceptable that the final decision be made by the patient or by the physician, if this was in line with their preference. Preferences as to who ought to make this final decision varied (i) between individuals and (ii) between decisions.

##### Preferences for decisional control vary between individuals

Some patients preferred to make the final decision (patient chooses), some wanted the decision‐making process to be evenly shared (shared choice), while others preferred to delegate final decisions to the doctor (patient delegates) (see Table 3). Importantly, when patients expressed wanting to delegate the decision to the physician, they noted that they would still have to consent to this final decision. All patients stressed the importance of being informed of treatment options and of being involved in the deliberation about different decisions.

**Table 3:** Preferences for decisional control varied between individuals^a^

Patient delegates	Shared choice	Patient chooses
**P38: …**when we reach a way of treatment I prefer to be mostly doctor‐directed but with my involvement so I understand, what is this single or multiple treatment…are there several? Which are the benefits? Which are the downsides…and so on and so forth. Pretty much I feel the more interactions you can have the better. You know? **I: Why do you say the doctor should make the final decision?** **P10:**Well, it's certain that it will be with my consent, but I have so much confidence in the physician. I don't have the tools to go beyond the information that I have; he has maybe the more broad technical information. Where he goes, in the end, he will always suggest to me one path or another, and if I don't want it, I think the treatment won't happen. But I have confidence in the medical information, I might not agree with the path because it's scary, for examples the electrodes in the brain, or the medications, but I will give him the benefit of the doubt to make these types of decisions, and I will accept after information.	**P14:** I would expect the doctor and I, and my spouse, to be involved as a team **P32:** I think the physician lays out your options and I think that it's up to you both to decide whether this would be best, or better for you, whether it is medication or doing some form of exercise, or climbing mountains or whatever it is. If the options are presented to you then you can both discuss the pros and cons and make (…) an informed decision. **P47:** Well I think that it should be done together. The doctor works based off of what he perceives from the patient, and it's in talking with the patient that he can learn even more. And that's why, if the patient gives frank and detailed information, well it certainly has to help the guide the doctor to the best solutions for the patient in question.	**P26:** The ultimate decision should be from the patient. **I: So why do you say that?** **P26:**Because he or she is the one who is suffering. They know what they are going through and they should take a chance on anything they want. It's not the doctor. **P26**′**:** Yeah, and I think ultimately it's, you know, it is the life of the person, you know? It should be…yeah. If the person, the patient has all his mental capacity then I think it should be…as long as it is…yeah…healthy mind. **P25:** Oh, the patient being involved in decision making, you're involved in everything. It's your life. I mean, it's not up to…I don't think it's up to…well, it's your decision but it should be discussed with your doctor and you. **P45:** I think that it comes back to the patient in the end. But after a discussion with the doctor that is sufficiently in depth, if you will. I think that the doctor has to be there to guide the patient to make his own decision. […] So I think that it's up to the physician to be a bit of a guide, and to try and see when he thinks the patient is headed towards the decision on his own.

a Note we use a spectrum of shared decision making, where responses under “shared choice” indicate an approximately even contribution between physician and patient to the health‐care decision, responses under “patient delegates” indicate the patient prefers the physician to make the final decision, and “patient chooses” indicates the patient is more likely to make the final decision. Individuals did not necessarily adhere to one preference for all decisions.

##### Preferences for participation vary between decisions

Individuals modulated their decision‐making preference based on the decision to be made. For example, some patients preferred that decisions about medication (e.g. dosage) be made by the doctor (patient delegates). For other decisions, such as decisions on treatments with potentially severe effects on the quality of life, patients wanted to play a bigger role in the decision‐making process (patient chooses). Relational context also affected their decision‐making preferences (e.g. an established relationship of trust with a physician may be necessary before delegating a decision). Thus, preferences for involvement in decision making are decision‐dependent and context‐ and relation‐dependent (see Table 4).

**Table 4:** Examples illustrating that preference for involvement in decision making are decision dependent and context and relation dependent

Decision‐dependent	Decisions requiring technical knowledge (e.g. medication) are often delegated to the physician: P9: I trust my doctors and I appreciate their treatments, and they are making a lot of decisions. They consult me and they tell me what they are doing. […] I'm not saying “no I want a lower dosage or a higher dosage”… I trust her on that. She's making that kind of decision. P10: The final decision on whether or not to increase the medication, it's him [the physician] that makes that decision, that's certain. But I like to know the reasons as to why he's making these decisions. P16: (…) for things like doses, I can't regulate that, he has expertise I will never have. […] So, there are certain decisions that he has to take because I need it. Decisions about lifestyle require patient involvement: P16: (…) the style of living, the way you need to live your life, that's up to you. You can help me by saying be careful, you know. You may eventually get to the point where you're going to trip and fall, so yeah if you're thinking about changing house, good for you. I think it's a good wise decision but the ultimate decision will be up to me. Physician has expertise about treatments; patient can decide overall treatment goals: P32: I think…as far as medication treatment, I believe the physician should make the ultimate decisions because he knows more about what effects it has and if it can counter attack whatever is the problem at the moment, but in the long run, we have to decide for ourselves what we're going to do, whether we're going to take that chemotherapy or not…and so on, but as I said before, it has to be an informed decision and listen to what the physician has to tell you…and decide together.
Context‐ and relation‐dependent	Personal and contextual factors impact decisions: P24: How old would I be? What would be my income? Where will I live? All of those are factors that I have that are outside of my [control] but I have to be taking care and into consideration before I make my decision and that's outside the doctor and medical care. Personal relationships impact decisions: P14: My spouse has a big influence on me. I used to be the one that took charge, and now the roles are reversed now that we are in our 70s and I very much respect her advice. Trusting relationship with physician is necessary for decision making: P60: It's their body. It's their life so I think the ultimate decision should be the patient's but taking into account that the doctor has said and bearing in mind whether you trust the doctor or you don't. If you don't trust the doctor you shouldn't be there in the first place. If you do trust the doctor your decision has to be in line with that knowledge…that your doctor would not suggest anything that would be harmful to you. You see? But basically the ultimate decision begins with the patient who has to sign these consent forms, not the doctor. The patient has to realise the doctor has gone to school and has had many more years of practice experience and to trust them.

##### Navigating decision making when there is disagreement

In a hypothetical situation of conflict (e.g. a patient and doctor cannot agree on a treatment), there was large support for the patient to be the final decision‐maker. However, many patients described how they would prefer to come to an agreement with their physician. In instances where an agreement cannot be met, some described the need to seek second opinions. In a minority of cases, patients were willing to follow the doctor's suggestion, provided that they have been sufficiently informed and they trust their physician.

##### Perspectives on patient exclusion from decision making

Allowing the physician to decide entirely which treatment is appropriate or excluding the patient from health‐care decision making was viewed as acceptable only when the patient is mentally incapable (i.e. patient lacks capacity). Some participants mentioned the lack of patient education or experience in medical encounters, the severity of disease or the complexity of treatment as situations that may warrant the physicians taking greater control. However, patients invoked these situations as hypothetical examples and generally did not associate these situations with themselves. Overall, patients wanted to be involved in decision making, even if only for information exchange.

#### 3.2.3 Patient perspectives on the patient–physician relationship in relation to decision making

Patients emphasized the importance of the patient–physician relationship, and they described the need to be respected as persons. Communication was the underlying theme in articulating the values important to their care (e.g. candour, honesty, understanding and empathy). For the patient–physician relationship, patients valued mutual respect, trust, openness and time for communication. The need for the relationship to be non‐hierarchical was noted.

#### Desired qualities of a physician

Patients described a desire for physicians to possess technical skills (e.g. give appropriate information, evaluate how the PD has progressed, be up‐to‐date) and interpersonal skills (e.g. listen, be empathetic, understanding). Central to both the technical and interpersonal skills was the importance of informed and sensitive communication. Indeed, communication was the chief concern, and other important physician qualities such as sincerity, caring and empathy were tied to communication skills.

#### Ideal qualities of a patient

Participants described various qualities that patients should possess, such as being open, honest and proactive. They also described the responsibilities a patient must take on, including self‐management practices, informing the doctor of new symptoms, preparing questions for the clinical encounter, seeking information from external sources, listening to the medical advice and complying with the agreed course of action or informing the physician if they choose to not follow the plan.

#### Patients and clinicians bring unique knowledge to the decision‐making encounter

Participants recognized that each agent in the patient–physician relationship possess different types of knowledge central to decision‐making processes (see Box 1). The physician was viewed as having the technical, specialized knowledge, based on their education and experience. Patients viewed this specialized knowledge as setting the neurologist apart from other care providers, making them an invaluable part of their care. At the same time, patients recognized that they possessed the knowledge of the lived experience of the disease, as well as awareness of their own values and goals for care.

Box 1 **Illustrative examples: each agent brings different types of knowledge to decision‐making processes**

1 Patient brings experiential knowledge:

P16: I value the doctor's opinion a lot as long as he values my opinion as well, because I'm the one that's living the disease. He might know about it but he doesn't have it. (…) I will always tell you, you don't know what it feels like until you've actually lived it. And [the specialist], there's no way that he can actually honestly and truly deep down inside know how I feel until he's in my shoes and he has Parkinson's. He can know tons of knowledge about it and that's what I respect about him, is his knowledge, but until he can get into my shoes and live with Parkinson's, that's where I come in, to kind of complement his studies.

Patient brings bodily experience:

P25: Well of course the doctor has more medical experience but the individual is the person having the bodily experience, you know all the problems that come with it, so of course they have to communicate with one another.

Physician brings medical knowledge:

P24: Well, basically the doctor has the capacity to evaluate based on the facts, based on the tests, based on everything, her experience and the medication and her training. She can tell me what she thinks is the best and from that time I will talk with her, ask questions, decide about it and together we'll plan for the future. (…) I'll follow her because I trust her. Because I know that she won't propose something for nothing. She'll propose some things because I may need it. With her experience, her know how and her past cases, if it's time for me to take medication.

Patient can undertake active information seeking; physician, active listening:

P38: As a patient you should get your hands on as much more specialist information as you can. Try to digest it and write down your questions and refer to ask them, so that's what I did. On the side of the doctor, I prefer the doctor to listen to me and to listen to all the symptoms that I might be able to describe and try to have the diagnosis as early as possible.

#### 3.2.4 Patient preferences for accessing information relevant to decision making

##### Utilized sources and types of information patients seek

Patients most commonly cited seeking PD information from the Internet (N=16), PD foundations or associations (N=12) and the medical personnel at the specialist clinic, including the neurologist and nurse clinician (N=10). Patients also accessed information from books (N=6), personal networks (e.g. family, friends, support groups; N=6), television, radio or newspaper (N=2), and from other sources such as conferences or specialized rehabilitation centres (N=3). The types of information patients wanted varied with sources (see Table 5).

**Table 5:** Types and sources of information sought about Parkinson's disease

Types of information	Source^a^	
	Clinic	Other
Assessment of PD state	13	0
Progression of PD	10	4
Treatments for PD	8	6
Scientific research related to PD^b^	5	6
PD symptoms	4	7
Self‐management strategies	4	6
Causes of PD	3	1
Complementary and alternative medicine	2	2
Other	1	0

a Research reports and opportunities for research participation.

b Numbers indicate the number of participants reporting the use of this source of information.

In the medical encounter, patients sometimes expressed a difficulty in knowing which questions to ask. In general, they were particularly interested in an assessment of the state of their PD, what to expect in terms of future progression of their illness, and treatment options. This was in line with the primary role patients expected from their physicians, which was to continually assess their condition and control their symptoms with medication as needed. In consulting other sources, patients wanted to learn most about PD symptoms, current and upcoming PD research, treatment options and self‐management strategies.

Different sources of information were viewed as having various advantages and disadvantages. Foundations were viewed as a reliable, focused, readily available source. Medical personnel were viewed as an expert and personalized source, but the time between appointments meant that they were not a readily available source of information. The Internet was often used to connect patients to foundations, to confirm information learnt elsewhere and to investigate information related to PD. However, the unclear reliability of some websites and the uncontrolled nature of the readily available information on the Internet were viewed as disadvantages. In particular, the detailed information on the most advanced stages of the disease was described as upsetting to some patients, leading some to limit their online research. About half of the patients described different strategies they use to distinguish between the reliability of the sources (e.g. using known sources such as foundation websites, or scientific and medical websites; checking if multiple sites gave the same information; remaining sceptical of unverified sources). At least a quarter of patients did not describe any strategies for reliability; they “just put in Parkinson's disease” and click on “whatever comes up” (P51).

Some patients complained that some sources (e.g. a video from a foundation depicting various exercises for PD) only represented elderly PD patients. This was viewed negatively as it did not represent their experience, and is important to note in the light of the average age of patients with PD (early to mid‐60s).^23^ The uncertainties of PD, including its cause, an individual's expected progression and the lack of objective tests for diagnosis, presented a challenge for some patients with PD. Patients struggled with wanting to know this information, despite its unavailability. The extent to which they understood that this information does not exist was unclear.

#### Limits to learning PD‐related information

Many patients expressed wanting to know as much information about PD as possible. Information about PD, especially about its progression, was viewed as important for the patient to adapt and prepare for the future. Patients acknowledged that the information can be upsetting, but felt that it was important for them to know, and that they should be able to adapt to the news, even if it was difficult. Despite multiple patients’ expressions of wanting to know everything, they also reported a limit either to what they wanted to learn or to what they wanted to focus on (see Box 2). In part, this was a practical concern, as many acknowledged the outcomes of the disease are uncertain and as a result many expressed a “take things as they come” attitude; a focus on the negative outcomes can be emotionally taxing. At the same time, most patients have a general knowledge of the long‐term outcomes of PD; this is generally learnt in the first few years after diagnosis, and as they adapted to their diagnosis, patients tended to focus on this less. At least one patient did not want to receive any information about PD, due to finding it emotionally distressing, and preferred that their partner receives the information instead. However, this patient expressed that they would not want important information to be hidden from them.

Box 2 **Patient preferences for information**

1 Patients express wanting to “know everything”:

P25: I need to be told everything that needs to be told, good or bad. […] I think the doctor needs to be totally honest.

P31: I would always want to have a chance to know something, even if it was really scary and really painful.

Patients note limits due to the unknowns of PD:

P31: I don't know that anybody has a crystal ball that can predict how I will turn out. So I just don't want to waste time thinking about… Not that I don't accept it, but how much is it worth devoting time talking about what are the eventual possibilities if they may not happen (…) I think I'm more practical about what is happening, how can that be addressed?

Adaptation to diagnosis can affect information preferences:

P31: I have a big, busy job. It's more than full time. I have a family that's very active, and I'm very busy with them. […] And I have lots of friends and lots of stuff going on, so I think there is a limit to how much I want to hear and invest in Parkinson's. When I first got the diagnosis, I was reading more, always from good sources. I was thinking about it more. I was writing things down about what I thought, but very naturally, it sort of assumed less of a prominent position. It's like, “Okay, yeah, you got Parkinson's. So what else are you doing?” Whereas, for a little time, it was really everything I was thinking about.

Emotional sensitivity can preclude desire for information:

P44: I don't want any [information]. I want [my spouse] to get it all. […] Because I'm frightened of what might happen…

## 4 Discussion

The results of this study are consistent with prior research showing variation in patient preferences.^13^, ^24^, ^25^ Importantly, our research is the first, to our knowledge, to qualitatively and quantitatively investigate health‐care decision‐making participation preferences in a chronic neurodegenerative disease population.

We found that most patients with PD describe wanting a kind of shared decision making, especially as this relates to information exchange and deliberation, while preferences for decisional control depend on the decision type (e.g. medication versus lifestyle) and on contextual and relational factors (e.g. age, income, need for trust in patient–physician relationship). Results from the API complement these qualitative observations. The average decision‐making score for participants was 63, which indicates a mid‐range preference for autonomy that can correspond to shared decision making. A detailed look at the API decision‐making scale results suggests that in some contexts or situations patients wanted less autonomy in medical decision making. In particular, patients with PD had lower autonomy preferences when it came to making a decision about when their next appointment should be, which may be a preference that reflects their actual experience. They also preferred that the physician takes greater control as their illness worsens, which may be connected to the types of impairments that can occur in late‐stage PD (e.g. dementia) and the relationship patients expect to develop with their physician over the course of their illness. The latter finding is in line with our qualitative data that suggest patients would find it acceptable to be excluded from decision making only when they were cognitively unable to do so. However, it contrasts with the findings from the PD‐focused vignettes in the API, where significantly more autonomy was desired as the disease progresses, and the most autonomy was desired when emotional symptoms were involved. On this last point, it is possible that emotional symptoms are perceived differently than motor or cognitive symptoms, and thus, patient preferences for autonomy differed specifically with this set of symptoms. The data gathered from the vignettes also suggest that patient preferences for autonomy differ in the general medical context, where patients wanted higher autonomy, versus in the specific PD care context. Survey results also revealed a trend for participants with lower levels of education (a professional college education or less) to have lower scores on the decision‐making scale of the API, than participants with higher levels of education (graduate or bachelor's degree). The difference observed did not reach statistical significance, but is in line with other research that suggests higher education is associated with higher preference for autonomy.^15^ These findings suggest that context is a complex modulator of autonomy preferences.

We found that most patients want full information about their condition and treatment options, which is consistent with prior research (e.g. see^26^, ^27^, ^28^). However, our data demonstrate why, in the context of a chronic neurodegenerative illness, patients might have reasonable limits to the types and amounts of information they want to know or focus on (e.g. due to the uncertainty in prognosis of PD, adaptation to the diagnosis and life with a chronic degenerative illness).

We also explored the importance of the patient–physician relationship and found that patients highly valued this relationship. For an excellent patient–physician relationship, they emphasized the importance of communication and, in particular, cited the need for physicians to possess strong interpersonal skills and for patients to take on certain responsibilities in their care. Their emphasis on the “human” side of interactions corresponds to the central aim of PCC to treat patients as persons.

### 4.1 Patient preferences for involvement in decision making are dynamic and support shared decision making

Patients’ preferences for involvement are not static; rather, they shift depending on decisions, context and relationships. This suggests a need to understand decision making in a more dynamic way.^29^, ^30^ Interestingly, patient preferences for involvement in information exchange and deliberation are more or less consistent, with the variation lying in desire for decisional control. This is noteworthy as there is some debate as to the extent to which each of these stages must be shared in order for the process to be considered shared decision making.^31^ The ease with which patients express a preference for information exchange and deliberation that is not mirrored when they are asked about preferences for decisional control may be related to the chronic nature of PD, where medically relevant decisions are not as discrete as they may be in more acute illnesses. For the patient with PD, certain decisions can necessitate different levels of involvement. For example, decisions about medication may require patient–provider partnership, patients may prefer to be more self‐directed in decisions about long‐term preparation for the disease and self‐management, and progression of the disease may require the physician or surrogate to assume greater decisional control over time. Articulating preferences about involvement in decision making can be challenging when there are a variety of decision types which might require different levels of involvement. This points to a need for clinicians to assess patient preferences for involvement on an on‐going basis, similar to recommended practices for evaluating decisional capacity.^32^ Tools to facilitate this evaluation and patient involvement in decision making may need to be developed.

Of interest, patients in our study specifically described many of the elements and qualities of shared decision making identified in a systematic review.^33^ They expressed a desire for essential elements of shared decision making, such as explanation of the problem, discussion of the pros and cons and explication of patient values and preferences; ideal elements such as mutual agreement; and general qualities, such as mutual respect, patient participation and partnership. Overall, our study provides empirical support for the relevance of shared decision making to patients in PCC. This is noteworthy considering shared decision making has been referred to as the “pinnacle” of PCC.^34^

### 4.2 Support for relational autonomy in patient‐centred care

Patient‐centred care, by respecting and responding to the wants, needs and values of patients, seeks to support the patient in decision making and thus needs to adopt a model of autonomy that promotes involvement of the patient, but does not leave them without support. PCC, in treating the patient as person, acknowledges that patients are complex, social beings, with interdependencies and interconnections that can influence decision making, a view that corresponds to “relational” or “contextualized” autonomy.^35^, ^36^, ^37^ This perspective on autonomy contrasts with some narrower understandings that interpret autonomy as conceding to individualistic decision making without due consideration for the social determinants of choice, or for the commitment to care and beneficence of health‐care providers.

The central tenet of relational autonomy is that “persons are socially embedded and that agents’ identities are formed within the context of social relationships and shaped by a complex of intersecting social determinants, such as race, class, gender and ethnicity.”^38^ This concept stems chiefly from feminist ethics, but also from pragmatist ethics,^39^, ^40^ and recognizes the effects of contextual and relational factors on decision making.

Our findings suggest that relational autonomy corresponds to how patients would envision their participation in decision making. Patients recognized the impact of contextual and relational factors on their involvement in decision making and they acknowledged the central role the patient–clinician relationship plays in their care. By explicitly adopting relational autonomy in the provision of PCC, clinicians are called on to recognize the different factors that can affect a patient's desire to be involved in decision making, and to respond to these factors in such a way that empowers patients in relation to their wants, needs and values.

## 5 Limitations

There are several limitations to our study. It is a cross‐sectional study with a sample population limited to patients who had early‐stage PD; how preferences may change over time, and the effects of advanced stages of PD on decision‐making preferences are unclear. Patients came from a large urban area, were serviced by a university‐level health centre, in a specialized PCC clinic within a publically funded health‐care system; this demographic may not be reflective of the general PD population. In particular, while average age and gender were closely matched to averages for PD populations,^23^ our sample size had a high‐level education, which is important to note given the effects of education on decision‐making preferences.^15^ Patients self‐reported their participation preferences; future studies of how their self‐reported participation preferences compares to their actual participation preferences would be of interest.

## 6 Conclusion

This study suggests that patients largely prefer a shared decision‐making approach, while individual patient preferences for involvement can vary between persons and between decision types. Consequently, clinicians may need to evaluate patient preferences for involvement on an on‐going basis, and tools to facilitate both this evaluation and patient involvement in decision making may need to be developed. Specific adoption of relational autonomy by clinicians would complement this approach. Our study illustrates that attention must be given to patient perspectives and that communication is key in clinical relationships. Improved understanding of individual preferences could enhance respect for persons and autonomy and make for PCC that is truly respectful of individual patients’ wants, needs and values.

## Sources of funding

Funding for this work was provided by the Canadian Institutes of Health Research (ER (PI) and EB (co‐I)) and the Fonds de recherche santé – Québec (career award, ER; master's scholarship in partnership with Unité SUPPORT du Québec, NZ).

## Conflicts of interest

The authors have no conflicts of interest to report.

## References

[1] Parkinson's Disease Foundation . Statistics on Parkinson's 2015 [accessed May 24 2015]. http://www.pdf.org/en/parkinson_statistics.

[2] Van Der Eijk M , Faber MJ , Al Shamma S , Munneke M , Bloem BR . Moving towards patient‐centered healthcare for patients with Parkinson's disease. Parkinsonism Relat Disord. 2011;17:360–364. 21396874 10.1016/j.parkreldis.2011.02.012

[3] Institute of Medicine (US) . Committee on Quality of Health Care in America. Crossing the Quality Chasm: A New Health System for the 21st Century. Washington, DC: National Academies Press; 2001. 25057539

[4] Canadian Medical Association . Achieving Patient‐Centred Collaborative Care. Ottawa: Canadian Medical Association; 2008.

[5] Michie S , Miles J , Weinman J . Patient‐centredness in chronic illness: What is it and does it matter? Patient Educ Couns. 2003;51:197–206. 14630376 10.1016/s0738-3991(02)00194-5

[6] Stewart MA . Effective physician‐patient communication and health outcomes: a review. CMAJ. 1995;152:1423–1433. 7728691 PMC1337906

[7] Duggan PS , Geller G , Cooper LA , Beach MC . The moral nature of patient‐centeredness: Is it “just the right thing to do”? Patient Educ Couns. 2006;62:271–276. 16356677 10.1016/j.pec.2005.08.001

[8] Abudi S , Bar‐Tal Y , Ziv L , Fish M . Parkinson's disease symptoms—’patients’ perceptions. J Adv Nurs. 1997;25:54–59. 9004011 10.1046/j.1365-2648.1997.1997025054.x

[9] Buetow S , Giddings LS , Williams L , Nayar S . Perceived unmet needs for health care among Parkinson's Society of New Zealand members with Parkinson's disease. Parkinsonism Relat Disord. 2008;14:495–500. 18316229 10.1016/j.parkreldis.2007.11.011

[10] Nisenzon AN , Robinson ME , Bowers D , Banou E , Malaty I , Okun MS . Measurement of patient‐centered outcomes in Parkinson's disease: What do patients really want from their treatment? Parkinsonism Relat Disord. 2011;17:89–94. 20952243 10.1016/j.parkreldis.2010.09.005 PMC3034811

[11] Wressle E , Engstrand C , Granérus AK . Living with Parkinson's disease: elderly patients’ and relatives’ perspective on daily living. Aust Occup Ther J. 2007;54:131–139.

[12] Mulley AG , Trimble C , Elwyn G . Stop the silent misdiagnosis: patients’ preferences matter. BMJ. 2012;345:e6572. 23137819 10.1136/bmj.e6572

[13] Robinson A , Thomson R . Variability in patient preferences for participating in medical decision making: implication for the use of decision support tools. Qual Health Care. 2001;10(suppl 1):i34–i38. 11533436 10.1136/qhc.0100034.. PMC1765743

[14] Say RE , Thomson R . The importance of patient preferences in treatment decisions—challenges for doctors. BMJ. 2003;327:542. 12958116 10.1136/bmj.327.7414.542 PMC192849

[15] Say R , Murtagh M , Thomson R . Patients’ preference for involvement in medical decision making: a narrative review. Patient Educ Couns. 2006;60:102–114. 16442453 10.1016/j.pec.2005.02.003

[16] Charles C , Gafni A , Whelan T . Decision‐making in the physician–patient encounter: revisiting the shared treatment decision‐making model. Soc Sci Med. 1999;49:651–661. 10452420 10.1016/s0277-9536(99)00145-8

[17] Ende J , Kazis L , Ash A , Moskowitz MA . Measuring patients’ desire for autonomy: decision making and information‐seeking preferences among medical patients. J Gen Intern Med. 1989;4:23–30. 2644407 10.1007/BF02596485

[18] Patton M . Purposeful Sampling. Qualitative Evaluation and Research Methods. London: Sage; 2004:169–183.

[19] Hellström I , Torres S . A wish to know but not always tell–couples living with dementia talk about disclosure preferences. Aging Ment Health. 2013;17:157–167. 23171298 10.1080/13607863.2012.742491

[20] Doherty C , Doherty W . Patients’ preferences for involvement in clinical decision‐making within secondary care and the factors that influence their preferences. J Nurs Manag. 2005;13:119–127. 15720481 10.1111/j.1365-2934.2004.00498.x

[21] Green J , Thorogood N . Qualitative Methods for Health Research. London: Sage; 2013.

[22] Hsieh HF , Shannon SE . Three approaches to qualitative content analysis. Qual Health Res. 2005;15:1277–1288. 16204405 10.1177/1049732305276687

[23] Samii A , Nutt JG , Ransom BR . Parkinson's disease. Lancet. 2004;363:1783–1793. 15172778 10.1016/S0140-6736(04)16305-8

[24] Levinson W , Kao A , Kuby A , Thisted RA . Not all patients want to participate in decision making. J Gen Intern Med. 2005;20:531–535. 15987329 10.1111/j.1525-1497.2005.04101.x PMC1490136

[25] Nilsson UG , Ivarsson B , Alm‐Roijer C , Svedberg P . The desire for involvement in healthcare, anxiety and coping in patients and their partners after a myocardial infarction. Eur J Cardiovasc Nurs. 2013;12:461–467. 23303764 10.1177/1474515112472269

[26] Beisecker AE , Beisecker TD . Patient information‐seeking behaviors when communicating with doctors. Med Care. 1990;28:19–28. 2296214 10.1097/00005650-199001000-00004

[27] Cassileth BR , Zupkis RV , Sutton‐Smith K , March V . Information and participation preferences among cancer patients. Ann Intern Med. 1980;92:832–836. 7387025 10.7326/0003-4819-92-6-832

[28] Gerteis M , Edgman‐Levitan S , Daley J , Delbanco TL . Through the Patient's Eyes: Understanding and Promoting Patient‐Centered Care. San Francisco: Jossey‐Bass; 1993.

[29] Muller S , Walter H . Reviewing autonomy: implications of the neurosciences and the free will debate for the principle of respect for the patient's autonomy. Camb Q Healthc Ethics. 2010;19:205–217. 20226104 10.1017/S0963180109990478

[30] Racine E , Dubljević V . Behavioral and brain‐based research on free moral agency: Threat or empowerment? In Illes J and Hossain S (eds): Neuroethics: Defining the Issues in Theory, Practice and Policy, Second edition. Oxford: Oxford University Press; In press.

[31] Flynn KE , Smith MA , Vanness D . A typology of preferences for participation in healthcare decision making. Soc Sci Med. 2006;63:1158–1169. 16697096 10.1016/j.socscimed.2006.03.030 PMC1637042

[32] Etchells E , Sharpe G , Elliott C , Singer PA . Bioethics for clinicians: 3. Capacity. CMAJ. 1996;155:657–661. 8823211 PMC1335218

[33] Makoul G , Clayman ML . An integrative model of shared decision making in medical encounters. Patient Educ Couns. 2006;60:301–312. 16051459 10.1016/j.pec.2005.06.010

[34] Barry MJ , Edgman‐Levitan S . Shared decision making—the pinnacle of patient‐centered care. N Engl J Med. 2012;366:780–781. 22375967 10.1056/NEJMp1109283

[35] Entwistle VA , Watt IS . Treating patients as persons: a capabilities approach to support delivery of person‐centered care. Am J Bioeth. 2013;13:29–39. 10.1080/15265161.2013.802060 PMC3746461 23862598

[36] Ells C , Hunt MR , Chambers‐Evans J . Relational autonomy as an essential component of patient‐centered care. Int J Fem Approaches Bioeth. 2011;4:79–101.

[37] Racine E , Aspler J , Forlini C , Chandler J . Broadening the lenses on complementary and alternative medicines in preclinical Alzheimer's disease: tensions between autonomy and contextual determinants of choice. Kennedy Inst Ethics J. 2016. In press. 10.1353/ken.2017.0002 28366902

[38] Mackenzie C , Stoljar N . Relational Autonomy: Feminist Perspectives on Autonomy, Agency, and the Social Self. New York: Oxford University Press; 2000.

[39] Wolf SM . Shifting paradigms in bioethics and health law: the rise of a new pragmatism. Am J Law Med. 1994;20:395–415. 7618636

[40] Racine E . Pragmatic neuroethics: the social aspects of ethics in disorders of consciousness. Handb Clin Neurol. 2013;118:357–372. 24182392 10.1016/B978-0-444-53501-6.00030-5

