# Supplementary material for: Examining chronic care patient preferences for involvement in health‐care decision making: the case of Parkinson's disease patients in a patient‐centred clinic
Source: Health Expect. 2016 Sep 14;20(4):655–64. doi: 10.1111/hex.12497 (PMC5513015; doi:10.1111/hex.12497)
Supplement: Supplementary file 1 [file HEX-20-655-s001.docx]

**Investigating preferences for decision making process and expectations about care in Parkinson’s patients**

Principal Investigator: Dr. Eric Racine, Neuroethics Research Unit, Institut de recherches cliniques de Montreal

Project lead: Dr. Emily Bell, Neuroethics Research Unit, Institut de recherches cliniques de Montreal

Co-Investigators: Dr. Anne-Louise Lafontaine, McGill University, Natalie Zizzo, Masters Student, Neuroethics Research Unit, Institut de recherches cliniques de Montreal

QUESTIONNAIRE

With this survey we hope to better understand the degree to which patients want to be involved in decisions about their medical care and receive information about their illness and subsequent treatment options. We hope that your participation in this study will allow us to suggest ways of improving the care that patients receive to reflect their preferences.

Date of survey completion:_________________

Female 🞏 Age (please enter precise age, not a range): _____

Male 🞏

What is the highest level of school you have completed or the highest degree you have received?

🞏 Elementary school

🞏 Secondary

🞏 Professional

🞏 CÉGEP

🞏 Bachelor

🞏 Master

🞏 PhD

🞏 Other: _________

Most recent occupation: ______________ N/A

Do you have an informal caregiver? 🞏 Yes 🞏No

If yes, what is your relationship to him or her? _______________

This is your:

🞏 Second visit with the specialist 🞏 Third visit with the specialist 🞏 Other ______________

Time elapsed between referral and first appointment with specialist at the clinic:

🞏 0-3 months 🞏 3-6 months 🞏 6-12 months 🞏 >12 months 🞏 I don’t remember

Please indicate your agreement with the following items on a scale of 1-5, where 1 = strongly disagree and 5 = strongly agree.

|  | **1 (Strongly disagree)** | **2** | **3** | **4** | **5 (Strongly agree)** |
| --- | --- | --- | --- | --- | --- |
| The important medical decisions should be made by your doctor, not by you. |  |  |  |  |  |
| You should go along with your doctor’s advice even if you disagree with it. |  |  |  |  |  |
| When hospitalized, you should *not* be making decisions about your own care. |  |  |  |  |  |
| You should feel free to make decisions about everyday medical problems. |  |  |  |  |  |
| If you were sick, as your illness became worse you would want your doctor to take greater control. |  |  |  |  |  |
| You should decide how frequently you need a check-up. |  |  |  |  |  |

In this section of the survey you will be given five different scenarios and asked to answer three questions about each scenario. For each of the questions, who should make the decisions (“you”, “mostly you”, “the doctor and you”, “mostly the doctor”, “the doctor alone”)?

|  | **You** | **Mostly you** | **The doctor and you** | **Mostly the doctor** | **The doctor alone** |
| --- | --- | --- | --- | --- | --- |
| Suppose you developed a sore throat, stuffy nose, and cough that lasted for three days. You are about to call your doctor on the telephone. Who should make the following decisions? | | | | | |
| Whether you should be seen by the doctor. |  |  |  |  |  |
| Whether a chest x-ray should be taken. |  |  |  |  |  |
| Whether you should try taking cough syrup. |  |  |  |  |  |
| Suppose you went to your doctor for a routine physical examination and he or she found that everything was all right expect that your blood pressure was high (170/100). Who should make the following decisions? | | | | | |
| When the next visit to check your blood pressure should be. |  |  |  |  |  |
| Whether you should take some time off from work to relax. |  |  |  |  |  |
| Whether you should be treated with medication or diet. |  |  |  |  |  |
| Suppose you have visited your physician for a routine check-up of your Parkinson disease. Your condition has been stable and you require a renewal of your medication. Who should make the following decisions? | | | | | |
| When your next visit should be. |  |  |  |  |  |
| When you should be seen by an allied health team member (occupational therapist, physiotherapist, nurse, speech pathologist). |  |  |  |  |  |
| If you should change your medication dose or timing. |  |  |  |  |  |
| For the last 2 weeks you have been feeling that the medication isn’t working as well, and you have found it increasingly difficult to get on with your everyday activities. Who should make the following decisions? | | | | | |
| When you should be seen by the physician. |  |  |  |  |  |
| Whether you should change the amount or timing of your medication, or add a new drug. |  |  |  |  |  |
| Whether you should be seen by another member of the allied health team (occupational therapist, physiotherapist, nurse, speech pathology). |  |  |  |  |  |
| For the last few months you have been experiencing a greater degree of emotional distress. Although your motor symptoms are unchanged you are feeling increasingly stressed or anxious and this is causing you difficulty impacting on your work and family interactions. Who should make the following decisions? | | | | | |
| Whether you should make an appointment to be seen sooner by your physician. |  |  |  |  |  |
| Whether your family should take part in the consultation to discuss these concerns. |  |  |  |  |  |
| Whether you should seek additional specialized support (social worker, psychiatrist, psychologist). |  |  |  |  |  |

Please indicate your agreement with the following items on a scale of 1-5, where 1 = strongly agree and 5 = strongly agree.

|  | **1 (Strongly disagree)** | **2** | **3** | **4** | **5 (Strongly agree)** |
| --- | --- | --- | --- | --- | --- |
| As you become sicker you should be told more and more about your illness. |  |  |  |  |  |
| You should understand completely what is happening inside your body as a result of your illness. |  |  |  |  |  |
| Even if the news is bad, you should be well informed. |  |  |  |  |  |
| Your doctor should explain the purpose of your laboratory tests. |  |  |  |  |  |
| You should be given information only when you ask for it. |  |  |  |  |  |
| It is important for you to know all the side effects of your medication. |  |  |  |  |  |
| Information about your illness is as important to you as treatment. |  |  |  |  |  |
| When there is more than one method to treat a problem, you should be told about each one. |  |  |  |  |  |

Did anyone help you fill out this questionnaire? 🞏 Yes 🞏No

If yes, who? 🞏 Spouse 🞏 Caregiver 🞏Other: ______________
